# Methylation of the transcription factor E2F1 by SETD6 regulates SETD6 expression *via* a positive feedback mechanism

**DOI:** 10.1016/j.jbc.2023.105236

**Published:** 2023-09-09

**Authors:** Margarita Kublanovsky, Gizem T. Ulu, Sara Weirich, Nurit Levy, Michal Feldman, Albert Jeltsch, Dan Levy

**Affiliations:** 1 The Shraga Segal Department of Microbiology, Immunology and Genetics, Ben-Gurion University of the Negev, Be'er-Sheva, Israel; 2 The National Institute for Biotechnology in the Negev, Ben-Gurion University of the Negev, Be'er-Sheva, Israel; 3 Institute of Biochemistry and Technical Biochemistry, University of Stuttgart, Stuttgart, Germany

**Keywords:** protein lysine methylation, SETD6, E2F1, lysine methylation

## Abstract

The protein lysine methyltransferase SET domain–containing protein 6 (SETD6) has been shown to influence different cellular activities and to be critically involved in the regulation of diverse developmental and pathological processes. However, the upstream signals that regulate the mRNA expression of SETD6 are not known. Bioinformatic analysis revealed that the SETD6 promoter has a binding site for the transcription factor E2F1. Using various experimental approaches, we show that E2F1 binds to the SETD6 promoter and regulates SETD6 mRNA expression. Our further observation that this phenomenon is SETD6 dependent suggested that SETD6 and E2F1 are linked. We next demonstrate that SETD6 monomethylates E2F1 specifically at K117 *in vitro* and in cells. Finally, we show that E2F1 methylation at K117 positively regulates the expression level of SETD6 mRNA. Depletion of SETD6 or overexpression of E2F1 K117R mutant, which cannot be methylated by SETD6, reverses the effect. Taken together, our data provide evidence for a positive feedback mechanism, which regulates the expression of SETD6 by E2F1 in a SETD6 methylation–dependent manner, and highlight the importance of protein lysine methyltransferases and lysine methylation signaling in the regulation of gene transcription.

Protein lysine methyltransferases (PKMTs) catalyze lysine methylation, an increasingly important post-translational modification (PTM) that regulates various signaling pathways (1, 2). All PKMTs use AdoMet as a methyl donor to methylate their target, the ε-amino group of lysine residues in proteins resulting in a monomethylated, dimethylated, or trimethylated lysine (Kme1, Kme2, or Kme3) (3, 4). SET domain–containing protein 6 (SETD6) is a 53 kDa PKMT containing a catalytic SET domain and a Rubisco substrate-binding domain (5, 6), which is encoded on chromosome 16 (16q21). SETD6 was originally identified as a monomethyltransferase that methylates RelA (p65), a subunit of the NF-κB complex. This methylation was shown to suppress the activation of NF-κB target genes (7). Since then, SETD6 has been implicated in various biological processes, such as gene expression regulation, chromatin remodeling, and cell cycle progression (5, 7, 8, 9, 10, 11, 12, 13, 14, 15, 16, 17, 18, 19). SETD6 has also been linked to several developmental steps and pathological conditions. For example, SETD6 was shown to be essential for memory consolidation, regulation of gene expression patterns, and spine morphology in the rat hippocampus (20). SETD6-mediated monomethylation of BRD4 at K99 (10) was demonstrated to regulate human papillomavirus transcription, genome replication, and segregation by binding of BRD4 to the E2 protein (21). In diabetic nephropathy, a chronic complication of diabetes, downregulation of SETD6 protected the cells from apoptosis and mitochondrial dysfunction (21). Furthermore, SETD6 function has been associated with several cancer types (8, 22, 23). In bladder cancer, SETD6 is upregulated and promotes cell survival through the NF-κB pathway (23). In contrast, SETD6-mediated methylation of PAK4 inhibited cell migration and invasion in breast cancer (17). Because of these associations with tumorigenic hallmarks, SETD6 may serve as an attractive target for therapeutic intervention (8, 22, 23).

The E2F family of transcription factors (TFs) is an important downstream effector of the retinoblastoma tumor–suppressor gene product (pRB) and plays a crucial role in regulating cell-cycle progression. In addition, E2Fs participate in a wide range of biological processes, such as differentiation, mitosis and the mitotic checkpoint, DNA replication, DNA-damage checkpoints, DNA repair, and apoptosis (24, 25, 26). The E2F family consists of eight members, namely E2F1–E2F8, which share the highest degree of homology in their DNA-binding domain explaining their ability to bind to a unique E2F consensus sequence (27). However, experimental evidence suggests that different members of the E2F family regulate distinct yet overlapping sets of target genes (28, 29, 30). The specificity of E2F binding to an individual binding site can be influenced by the DNA sequence as well as interactions with other TFs that are bound to adjacent regulatory elements (27, 31).

The observation that E2F1 is closely involved in regulating cellular processes, which are also controlled by SETD6, suggested a potential functional crosstalk between SETD6 and E2F1. Here, we demonstrate that SETD6 monomethylates E2F1 at K117 *in vitro* and in cells. We further show that E2F1 methylation increases the occupancy of E2F1 at the SETD6 promoter, resulting in the increased expression of SETD6 mRNA. Together, our findings suggest a new mechanistic dimension for the selective regulation of SETD6 mRNA expression, which is mediated by E2F1 activity *via* SETD6-dependent E2F1 methylation in a positive feedback mechanism.

## Results

### E2F1 regulates SETD6 mRNA expression

**Figure 1:**
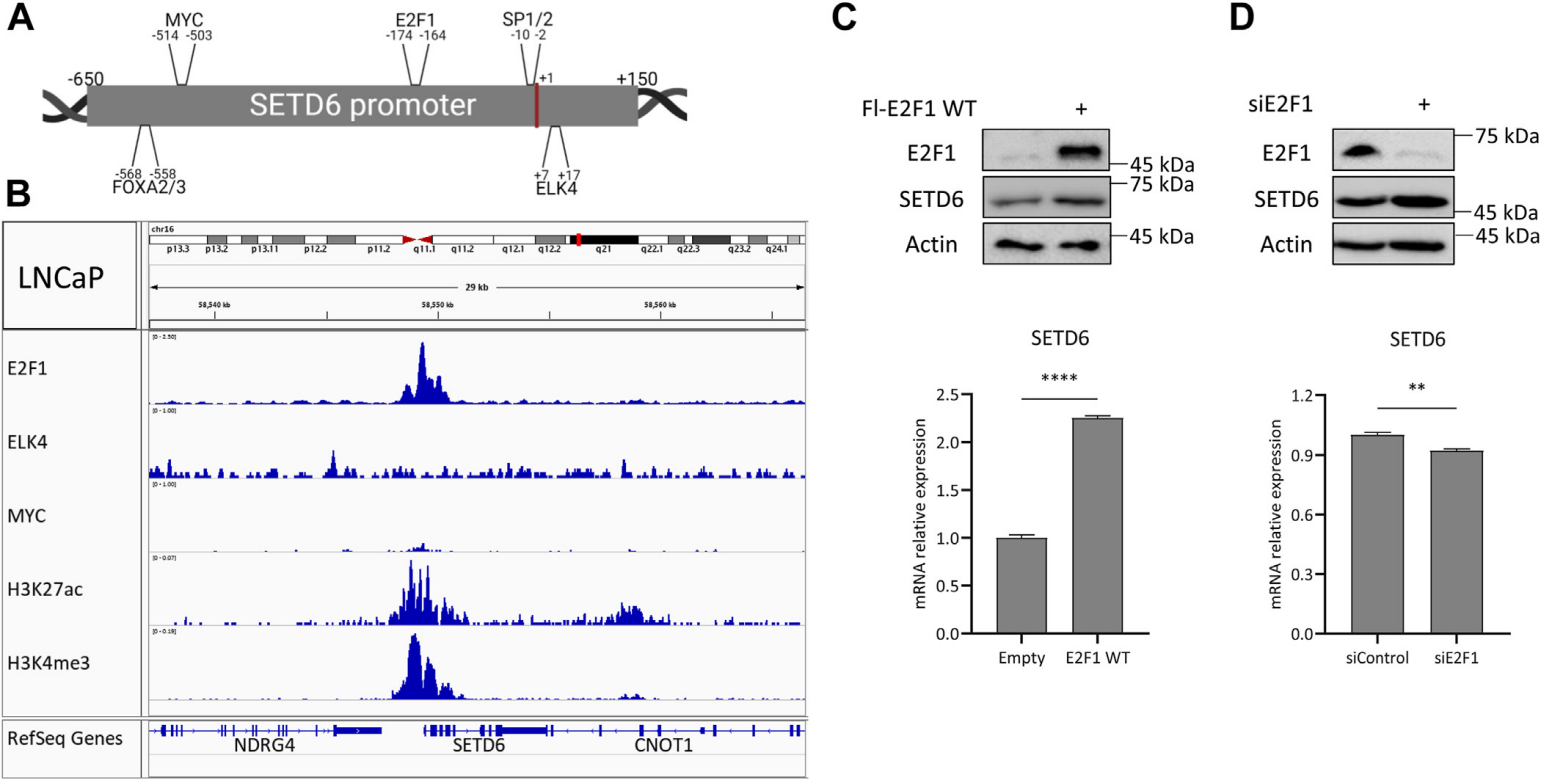
**E2F1 is predicted to bind at SETD6 promoter and regulates SETD6 mRNA levels.***A*, scheme of SETD6 promoter and several human transcription factor–binding sites that were predicted at the genomic region of the SETD6 promoter in the JASPAR database (https://jaspar.genereg.net/) with a relative profile score threshold of at least 90%. *B*, SETD6 promoter genomic region (*blue highlight*) with E2F1 (GSM1656410), ELK4 (GSM1424528), MYC (GSM1907203), H3K27ac (GSM1907213), H3K4me3 (GSM1907211) ChIP-Seq data in prostate cancer cell lines. Data were extracted by the Cistrome Data Browser (http://cistrome.org/db/#/) and visualized using the IGV software. *C* and *D*, DU145 cells were transfected with FLAG-E2F1 WT (*C*) or with siRNA specifically targeting endogenous E2F1 (*D*). About 24 h post-transfection, protein levels were assessed by WB (*top*) using the indicated antibodies, and the SETD6 mRNA expression levels were measured using RT–qPCR (*bottom*). mRNA expression levels were normalized to mRNA expression levels of GAPDH housekeeping gene. Error bars are SEM. Statistical analysis is based on five experimental repeats. ∗∗*p* ≤ 0.002 and ∗∗∗∗*p* ≤ 0.00001. ChIP-Seq, chromatin immunoprecipitation; qPCR, quantitative PCR; SETD6, SET domain–containing protein 6; WB, Western blot.

**Supporting Figure S1:**
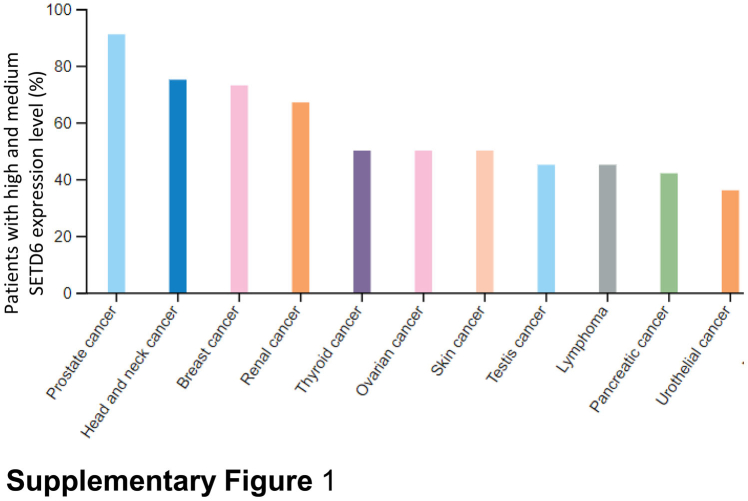
**Expression levels of SETD6 in various cancer.** The graphs were generated using the Human Protein Atlas resource (https://www.proteinatlas.org/). For each cancer, color-coded bars indicate the percentage of patients (maximum 12 patients) with high and medium SETD6 protein expression level.

Exploration of the Human Protein Atlas resource (https://www.proteinatlas.org/) summary on the pathology of SETD6 using immunohistochemical analysis showed that malignant prostate cells had the highest rate of SETD6 protein expression, compared with other cancerous tissues (Fig. S1). To investigate the potential mechanisms of *SETD6* gene regulation, we evaluated the presence of potential TF binding sites at the *SETD6* promoter region (https://jaspar.genereg.net/) (32). We identified 48 TFs that potentially bind at *SETD6* promoter (Fig. 1*A*, Table S1). To find out if these TFs are enriched in prostate cancer cells, we next searched publicly available chromatin immunoprecipitation sequencing (ChIP-Seq) databases (Gene Expression Omnibus [GEO] datasets: https://www.ncbi.nlm.nih.gov/gds, Cistrome Data Browser: http://cistrome.org/db/#/) for selected TFs. Interestingly, our analysis revealed selective enrichment of E2F1, ELK4, and MYC in prostate cells (Fig. 1*B*). Focusing on the promoter area of *SETD6*, which is in an open chromatin state marked by H3K27Ac and H3K4me3, we could not identify any enrichment for Myc and ELK4 but observed that E2F1 is highly enriched (Fig. 1*B*). We therefore decided to focus on the role of E2F1 in the expression regulation of SETD6 in prostate cells. Overexpression of FLAG-E2F1 in DU145 cells followed by quantitative PCR (qPCR) analysis revealed an increase of SETD6 mRNA expression levels (Fig. 1*C*). We confirmed these results by qPCR analysis subsequent to treatment of DU145 cells with siRNA targeting endogenous E2F1 (Fig. 1*D*). No change was observed in SETD6 protein levels corresponding to Western blot (WB) experiments in the investigated time scale. These results suggest that E2F1 stimulates SETD6 transcription.

### E2F1 activates SETD6 promoter transcription in a SETD6-dependent manner

**Figure 2:**
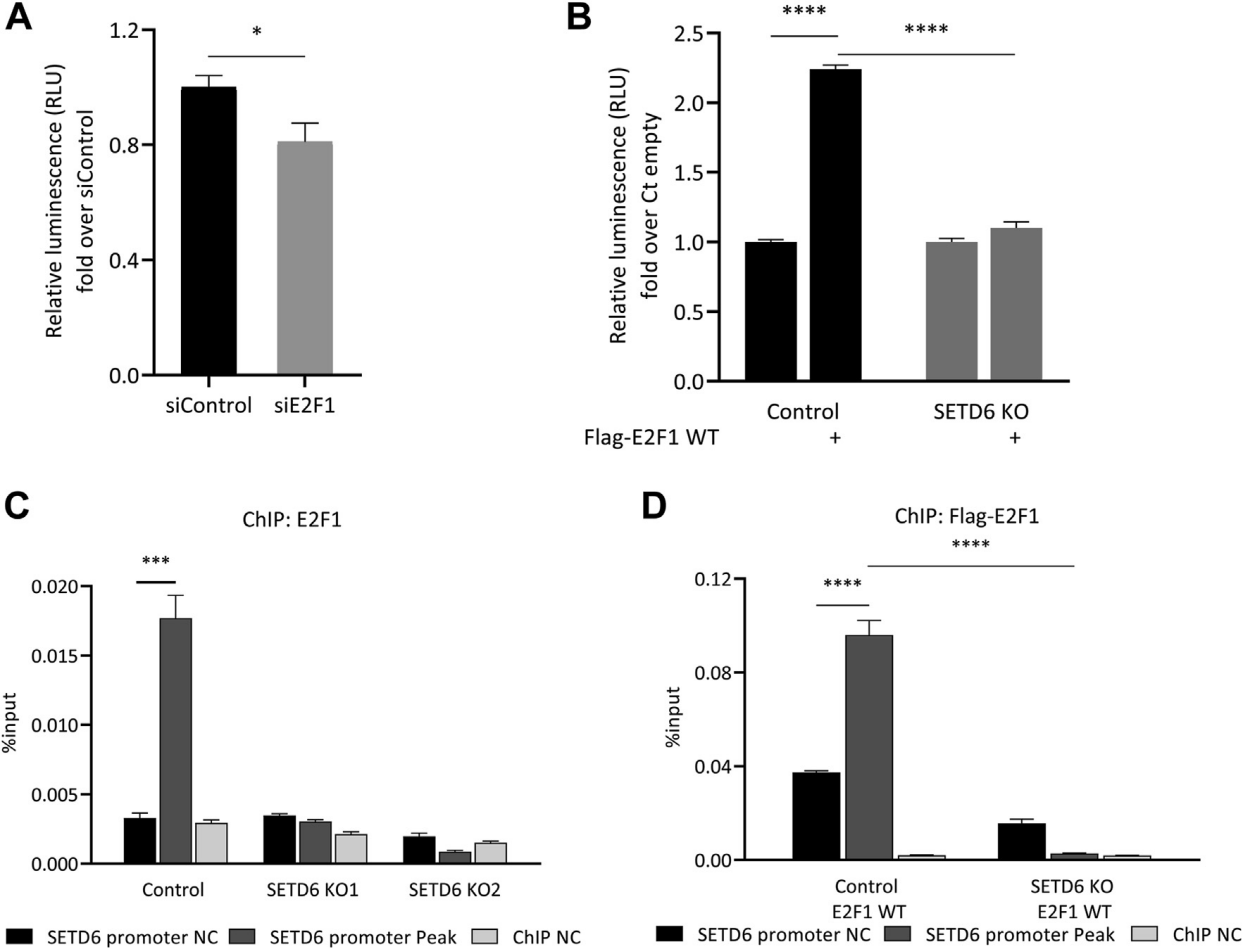
**E2F1 activates SETD6 transcription in an SETD6-dependent manner.***A*, dual-luciferase assay in DU145 cells transfected with siRNA targeting endogenous E2F and the full promoter region of SETD6 cloned to pGL3-plasmid (positions −650 to +150 from TSS). About 24 h post-transfection, the whole cell lysates were subjected to dual-luciferase assay (Promega). Relative luminescence was calculated after normalization of the firefly luciferase signal over Renilla luciferase control. *B*, same as in *A* with overexpression of empty or FLAG-E2F1 WT in control and SETD6 KO cells. Values are fold change over siControl. Error bars are SD. Statistical analysis was performed for three experimental repeats. ∗*p* ≤ 0.03 and ∗∗∗∗*p* ≤ 0.0001. *C*, chromatin immunoprecipitation (ChIP) assay in control and SETD6 KO DU145 cells (two independent SETD6 guide RNAs [gRNAs]). *D*, same as in *C* with overexpression of FLAG-E2F1 WT in control or SETD6 KO cells. About 24 h after transfection, the chromatin fraction of the cells was immunoprecipitated with endogenous E2F1 antibody (*C*) or FLAG antibody (*D*). The bound DNA was purified and amplified by quantitative PCR (qPCR) using specific primers to SETD6 gene promoter regions. SETD6 promoter peak (*dark gray bars*), peak that was identified in the ChIP-Seq experiments (Fig. 1*B*); SETD6 promoter NC, promoter region without a SETD6 noticed peak (NC), *black bars*; ChIP NC, distal NC region (*gray bars*). Graphs show the percentage input of the quantified DNA. Error bars are SEM. Statistical analysis was performed for four experimental repeats. ∗∗∗*p* ≤ 0.0002 and ∗∗∗∗*p* ≤ 0.0001. NC, negative control; SETD6, SET domain–containing protein 6.

**Supporting Figure S2:**
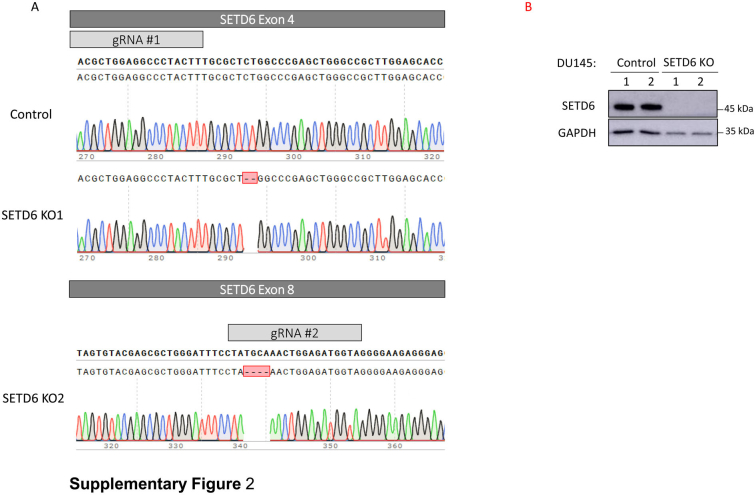
**SETD6 KO validation.** (*A*+*B*) DU145 control and SETD6 knock-out (SETD6 KO) were generated using the CRISPR/Cas9 system, following single-clone selection. *A*, chromatograms of Sanger sequencing of control cells and two SETD6 KO cells generated from two independent gRNAs targeting SETD6 exon 4 and exon 8. *B*, WB analysis with the indicated antibodies is shown for two single clones of Control and SETD6 KO cells.

To examine the possibility that E2F1 directly regulates *SETD6* transcription, we cloned the full-length promoter of SETD6 upstream to a luciferase reporter gene. Knockdown of E2F1 expression using siRNA in DU145 cells, followed by luciferase assay, confirmed E2F1 regulation of *SETD6* promoter activation (Fig. 2*A*). In a reciprocal experiment, we found a significant elevation in the promoter activity after overexpression of FLAG-E2F1 (Fig. 2*B*). Interestingly, activation of luciferase transcription was lost in SETD6 KO cells, even when accompanied by E2F1 overexpression (Fig. 2*B*). Sequence validation of the guide RNAs and WB is shown in Fig. S2, *A* and *B*, respectively. Collectively, these data suggest that E2F1-mediated activation of SETD6 transcription is potentially regulated in a SETD6-dependent manner.

To further test this hypothesis, we performed ChIP followed by qPCR analysis (ChIP–qPCR) to compare the occupancy of the endogenous E2F1 or overexpressed FLAG-E2F1 WT in DU145 control and SETD6 KO cells. ChIP–qPCR confirmed E2F1 binding to the specific region located in the SETD6 promoter correlating with the peak observed in ChIP-Seq data (Fig. 2, *C* and *D*). The enrichment of endogenous E2F1, as well as of the overexpressed FLAG-E2F1, was significantly lower at all the tested regions in the absence of endogenous SETD6. These data raised the hypothesis that SETD6 and E2F1 are linked and might have a functional cellular crosstalk between them in prostate cancer.

### SETD6 methylates E2F1 *in vitro* and in cells

**Supporting Figure S3:**
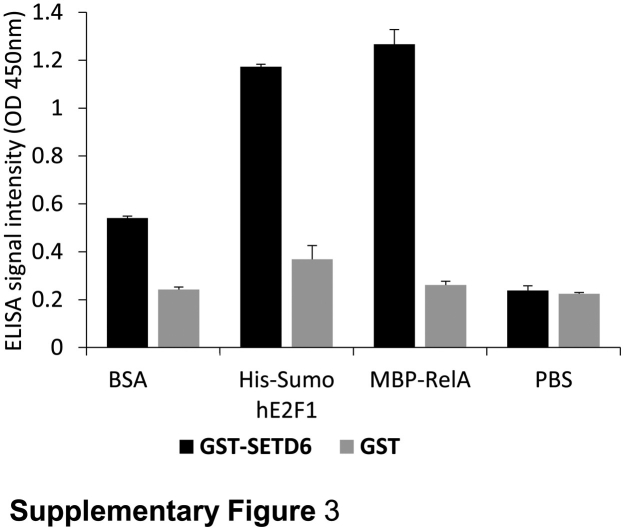
**ELISA assay showing the interaction between recombinant SETD6 and E2F1.** His-SUMO-E2F1, MBP-RelA or BSA were bound to the surface of a 96-well plate, incubated with GST-SETD6, or GST protein as negative control, and binding was analyzed with anti-GST antibody. RelA served as positive control for interaction with SETD6, while BSA served as negative control. PBS served as background control. Error bars are s.d. based on three experimental replicates.

We first investigated the physical interaction between E2F1 and SETD6 (Fig. S3). A direct interaction between the proteins was tested in an ELISA. In these experiments, recombinant His-SUMO-E2F1, MBP-RelA as positive control, or bovine serum albumin (BSA) as negative control were immobilized on a 96-well plate, followed by incubation with recombinant glutathione-*S*-transferase (GST)-tagged SETD6 or GST. A significant direct interaction was observed between SETD6 and E2F1. Given the enzymatic activity of SETD6 and its physical interaction with E2F1 *in vitro*, we hypothesized that SETD6 methylates E2F1.

**Figure 3:**
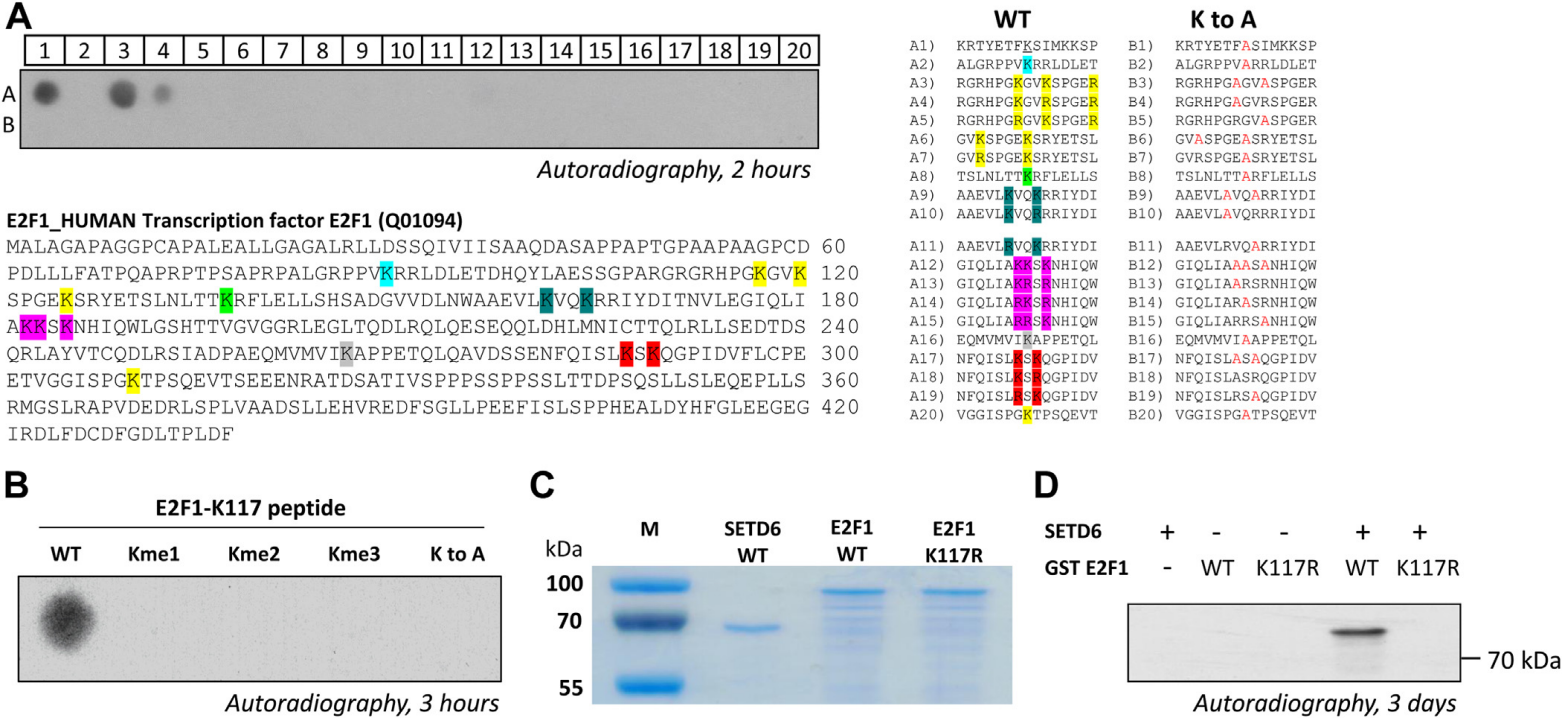
**SETD6 methylates E2F1 *in vitro* at K117 at peptide and protein levels.***A*, methylation of a peptide SPOT array for determination of the target lysine of SETD6 on E2F1. About 15-amino acid long peptides containing all lysine residues of E2F1 and variants in which individual lysine residues were replaced by alanine were synthesized on a peptide SPOT array and incubated with recombinant SETD6 in the presence of radioactively labeled AdoMet. The RelA peptide (in spot A1) served as positive control, whereas the mutated RelA K310A in spot B1 served as the negative control. Peptide sequences of the individual spots are presented on the *right*. *B*, same experiment as described in *A* was performed with a peptide array containing unmodified (WT) or modified (Kme1, Kme2, Kme3, and a K to A mutated) E2F1 K117 peptides (amino acids 111–125, same as in spot A3). *C*, Coomassie stain of purified His-SETD6, GST-E2F1 WT, and GST-E2F1 K117R mutant to show equal loading of E2F1 and E2F1 K117R substrate proteins used for *in vitro* methylation experiments. *D*, *in vitro* methylation assay of GST-E2F1 (WT and the mutant K117R) by recombinant His-SETD6 in the presence of radioactively labeled AdoMet. GST, glutathione-*S*-transferase; SETD6, SET domain–containing protein 6.

SETD6 potential enzymatic activity on E2F1 was first tested on peptide substrates. To this end, 15-amino acid long peptides were synthesized on a cellulose membrane using the SPOT technology (33, 34) to contain the 14 lysines within the E2F1 sequence and the corresponding K to A mutants. The RelA peptide (7) served as positive control. The peptide arrays were then subjected to an *in vitro* methylation reaction using recombinant SETD6 and ^3^H-AdoMet as the methyl group donor. Interestingly, we discovered a strong methylation signal on the E2F1 111 to 125 peptide containing residues K117 and K120 (spot A3). Spot A4 only containing K117 showed reduced methylation. The methylation signal was completely lost if the lysine in the peptide, corresponding to K117 in E2F1, was exchanged by alanine (spots B3 and B4), strongly implying that K117 is the primary methylation site of SETD6 in E2F1, and K120 supports the methylation (Fig. 3*A*). To validate this finding and to examine the state of methylation, we repeated the same experiments with unmodified (WT), Kme1, Kme2, and Kme3 E2F1 (amino acids 111–125) peptides. On this array, we only observed a methylation signal at the WT sequence but not in the modified peptides (Fig. 3*B*) suggesting that at the peptide level, SETD6 monomethylates E2F1 specifically at K117. To test if K117 in full-length E2F1 is methylated by SETD6, we cloned, expressed, and purified full-length WT and K117R mutant E2F1 (Fig. 3*C*). The *in vitro* methylation assay presented in Figure 3*D* demonstrates that SETD6 methylates E2F1 WT, but no signal was observed when the E2F1 K117R mutant was used. These results indicate that SETD6 methylates E2F1 specifically at K117 *in vitro*.

**Figure 4:**
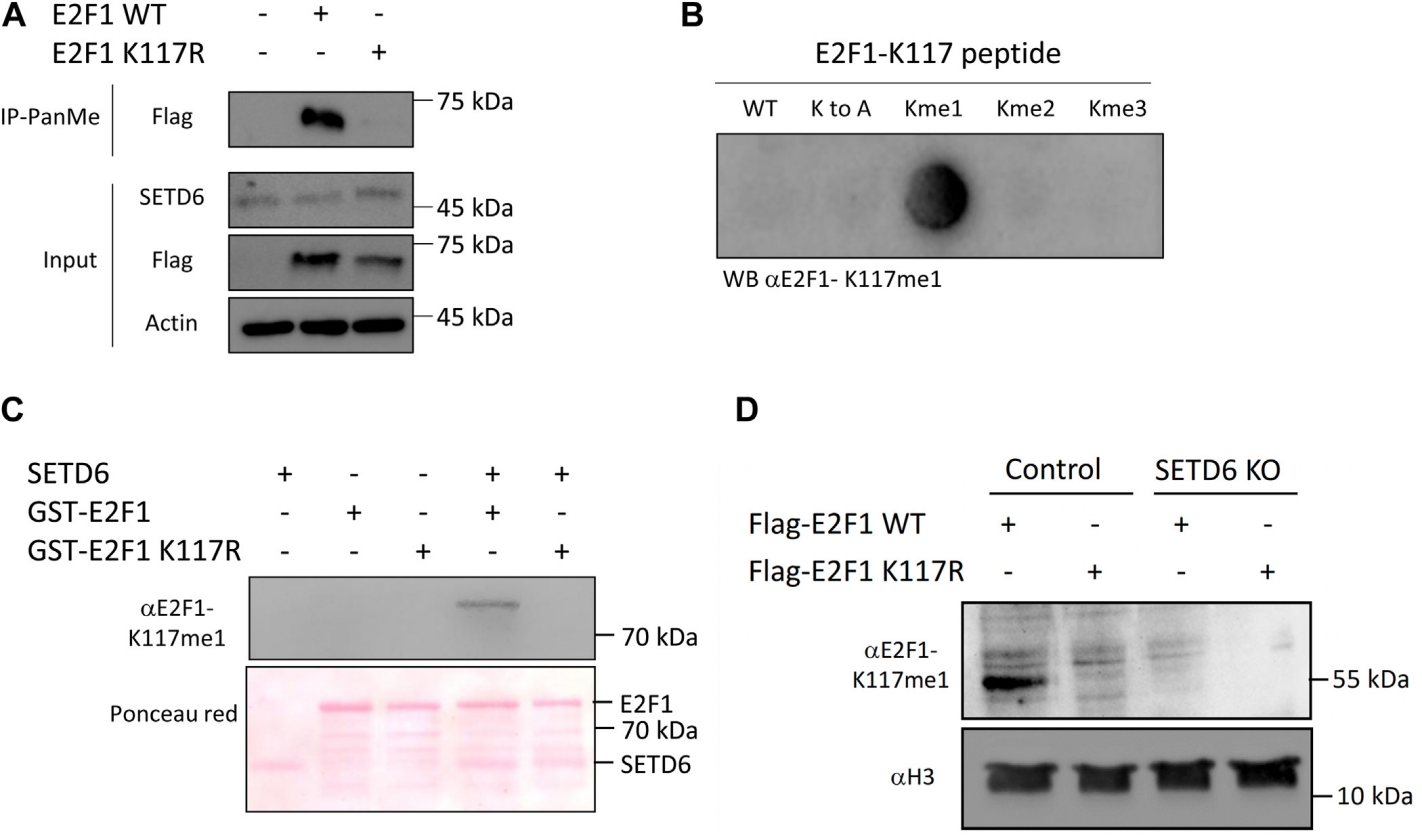
**SETD6 methylates E2F1 at K117 in cells.***A*, DU145 cells were transfected with FLAG-E2F1 WT or the respective K117R mutant. After 24 h, whole cell lysates were immunoprecipitated with Pan-Kme1 antibody, followed by WB analysis with indicated antibodies. *B*, for validation of the specificity of the custom-made E2F1-K117me1 antibody, a peptide SPOT array with 15-amino acid long unmodified (WT) or modified (Kme1, Kme2, Kme3, and a K to A mutated) E2F1 peptides (amino acids 111–125) was incubated with the antibody and binding analyzed by a secondary antibody and ECL. *C*, *in vitro* methylation assay GST-E2F1 (WT and the mutant K117R) by recombinant His-SETD6 using unlabeled AdoMet as cofactor. After methylation, WB analysis was performed using the E2F1-K117me1 antibody. Ponceau red staining was performed to visualize loading controls. *D*, DU145 control and SETD6 KO cells were transfected with the indicated plasmids. After 24 h, the whole protein lysate was analyzed by WB with the E2F1-K117me1 antibody. WB against H3 was included as input loading control. GST, glutathione-S-transferase; SETD6, SET domain–containing protein 6; WB, Western blot.

To investigate if E2F1 is methylated at K117 in cells, we immunoprecipitated overexpressed FLAG-E2F1 WT and the K117R mutant from DU145 using a pan-Kme1 antibody (Fig. 4*A*). While a strong pulldown was observed for E2F1 WT, the methylation signal of E2F1 K117R in DU145 decreased. We next purchased a custom-made site-specific antibody for E2F1 K117me1. Validation of the antibody specificity in peptide SPOT array binding experiments showed that it specifically recognizes monomethylated E2F1 K117, but not the unmodified, Kme2, and Kme3 peptides (Fig. 4*B*). We next utilized this antibody for an *in vitro* methylation assay with recombinant proteins showing that E2F1 WT but not the K117R mutant is methylated in the presence of recombinant SETD6 (Fig. 4*C*). We further found that SETD6 methylates E2F1 in control cells but not when the E2F1 K117R mutant was used or in SETD6 KO cells (Fig. 4*D*). Taken together, these results indicate that E2F1 is methylated by SETD6 *in vitro* and in cells, and lysine 117 is the primary methylation site.

### E2F1 methylation at K117 regulates SETD6 promoter activation

**Figure 5:**
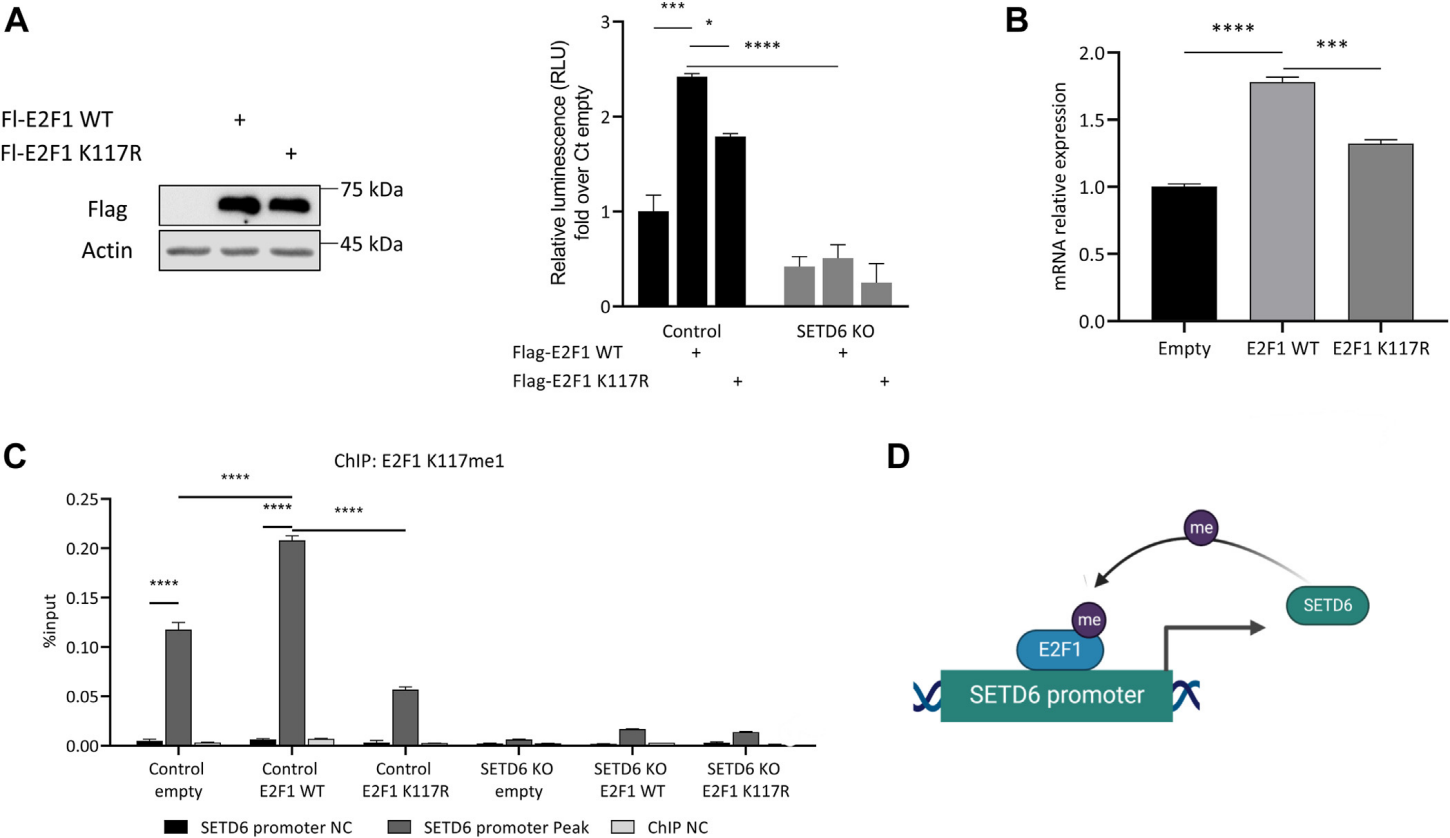
**E2F1 regulates SETD6 mRNA levels in an SETD6-dependent manner.***A*, dual-luciferase assay 24 h after transfection with empty plasmid, FLAG-E2F1 WT, or FLAG-E2F1 K117R mutant and the full-length SETD6 promoter luciferase construct in control and SETD6 KO cells. Values are fold change over empty control. E2F1 protein levels were assessed by WB (*left*). *B*, DU145 cells were transfected with empty plasmid, FLAG-E2F1 WT, or FLAG-E2F1 K117R mutant. SETD6 mRNA expression levels were measured using RT–qPCR. mRNA expression levels were normalized to mRNA expression levels of GAPDH housekeeping gene. *C*, chromatin immunoprecipitation (ChIP) assay 24 h after transfection with empty plasmid, FLAG-E2F1 WT, or FLAG-E2F1 K117R mutant. The chromatin fractions of the cells were immunoprecipitated with anti-E2F1 K117me1. Error bars are SEM. Statistical analysis was performed for five experimental repeats. ∗∗*p* ≤ 0.002, ∗∗∗*p* ≤ 0.0002, and ∗∗∗∗*p* ≤ 0.00001. *D*, graphical model of our findings. E2F1 methylation by SETD6 regulates the mRNA expression of SETD6 in a positive feedback mechanism. qPCR, quantitative PCR; SETD6, SET domain–containing protein 6; WB, Western blot.

Having demonstrated that E2F1 binds the SETD6 promoter and activates its transcription, we hypothesized that there could be a molecular feedback mechanism by which the methylation of E2F1 at K117 by SETD6 may affect the transcription of SETD6. To address this hypothesis, we first tested the activity of the recombinant SETD6 promoter driving luciferase expression in cells overexpressing E2F1 WT or E2F1 K117R mutant. A significant increase in the luciferase activity was observed in cells overexpressing WT compared with the K117R mutant (Fig. 5*A*). Consistent with these results, a significant elevation in endogenous SETD6 mRNA expression level was observed in cells overexpressing WT E2F1 compared with the E2F1 K117R mutant (Fig. 5*B*). To follow up on these results, we performed ChIP–qPCR experiments to test the SETD6 promoter occupancy by E2F1 in DU145 control and SETD6 KO cells overexpressing FLAG-E2F1 WT or FLAG-E2F1 K117R mutant, using the specific E2F1 K117me1 antibody for pulldown (Fig. 5*C*). The methylated E2F1 was identified more abundantly at the predicted region of SETD6 promoter in overexpressed FLAG-E2F1 WT DU145 control cells than in FLAG-E2F1 K117R or SETD6 KO cells (Fig. 5*C*). It is important to note that the E2F1 K117me1 antibody detects both endogenous and the expressed FLAG-tagged E2F1 if it is methylated at K117, and therefore, a signal is also observed in the control cells expressing E2F1 K117R mutant. These data provide evidence that E2F1 is present at the promoter of SETD6, and specifically, methylated E2F1 at K117 is more inclined to bind to this site. Collectively, our results suggest that methylated E2F1 binds to the SETD6 promoter and activates its expression in an SETD6- and a methylation-dependent manner (Fig. 5*D*).

## Discussion

In recent years, lysine methylation has been identified as an integral part of cellular biology and a key regulator of physiological and pathological processes in the cell. However, only a fraction of methylation events in the human proteome has been characterized. Here, we provide further insights into the lysine methylation field by the identification of E2F1 as a new SETD6 substrate, which in turn regulates the expression of SETD6 in a methylation-dependent manner. This SETD6-mediated methylation of E2F1 may have implications for the progression of diseases, including cancer. We may envision that the positive feedback mechanism, which is mediated by E2F1 methylation, is important for not only the tight regulation of SETD6 transcription but also SETD6 enzymatic activity to methylate other downstream substrates. Such a mechanism will ensure maintenance of the steady-state level of SETD6 to allow proper regulation of cellular pathways in which SETD6 is involved (7, 8, 9, 10, 11, 12, 15).

E2F1 activity has been shown to be regulated through extensive PTMs (35, 36, 37, 38). Interestingly, E2F1 is also subjected to acetylation (39, 40), arginine methylation (41), and NEDDylation (42) at K117, the exact same residue we have identified in this study to be methylated by SETD6. These modifications are located near the DNA-binding domain of E2F1. E2F1 is acetylated at K117, K120, and K125 in response to DNA damage (43). This modification was shown to increase E2F1 stability, DNA-binding affinity (39), and create a binding motif for the bromodomains of the p300/KAT3B and CBP/KAT3A acetyltransferases. Lysine residues 117, 120, 125, 182, 183, and 185 are required for efficient NEDDylation of E2F1 by ubiquitin-like modifier NEDD8. Experimental evidence suggests that K185 is particularly important for this PTM (42). NEDDylation results in decreased E2F1 stability, lower transcriptional activity, and slower cell growth. This specific PTM is regulated by SETD7-mediated methylation of E2F1 at K185, in response to DNA damage (44). This modification attenuates the level of E2F1 expression, by inhibition of acetylation and phosphorylation of the protein at distant residues and, simultaneously, by stimulation of polyubiquitination and subsequent degradation of E2F1 by 26S proteasome (35, 44). Yet, E2F1 forms a negative regulatory loop with SETD7, as it was demonstrated that SETD7 coactivates E2F1-dependent transcription of CCNE1 gene, thus promoting cell proliferation, through successful exit from the G1/S checkpoint arrest (45). While it can be assumed that methylation of K117 prevents other types of modifications at this residue, future studies are required to deeply assess if there are physiological and pathological links between these different modifications of E2F1 and the methylation of K117 by SETD6.

We have previously shown that BRD4 methylation by SETD6 at K99 regulates the recruitment of E2F1 to chromatin to selectively regulate the expression genes involved in protein translation. Once BRD4 is methylated, the recruitment of E2F1 to translation-related target genes is inhibited in a SETD6- and K99 methylation-dependent manner (10). Now, with the discovery that E2F1 is also methylated by SETD6, an intriguing working hypothesis that should be examined in the future is to test if E2F1 has to be methylated at K117 in order to control its association with the DNA and its genomic distribution.

Our results indicate that the methylation of K117 on E2F1 by SETD6 has an impact on E2F1's binding to the promoter region of the SETD6 gene, thereby modulating SETD6 gene expression. Specifically, methylation of E2F1 at K117 results in an increase in the enrichment of E2F1 at the SETD6 promoter and subsequently augments transcription of the SETD6 promoter, leading to elevated levels of SETD6 mRNA expression. We provide evidence for the existence of SETD6-E2F1 feedback loop. The characterization of feedback mechanisms have an enormous contribution for our understanding of cellular signaling pathways and network dynamics in many physiological and pathological scenarios (46). This include also feedback mechanisms that are mediated by E2F1 such as the NFKB (47) and the KRAS (48, 49) signaling pathways. Therefore, it is not unusual that methylation by SETD6 directs E2F1 transcriptional activity to regulate SETD6 mRNA transcription and may have a regulatory role to govern functional dynamics of cellular processes. While this work focuses on the role of E2F1 methylation in the regulation of SETD6 mRNA expression, one can speculate that modulation of the transcriptional effects of E2F1 by methylation at K117 is not restricted only to the SETD6 promoter but may have more broader roles in selective regulation of global gene expression programs. This hypothesis should be tested in the future using multiple genomic approaches, such as RNA-Seq, ChIP-Seq, and ATAC-Seq.

The TF E2F1 has many roles in the regulation of diverse oncogenic-related cellular pathways and phenotypes in several types of cancers (50, 51, 52, 53). In prostate cancer specifically, E2F1 was shown to act in a dichotomic manner in several oncogenic processes (54). However, the regulation of E2F1 transcriptional activity is still poorly understood. In this article, using biochemical and molecular biology approaches, we discovered how methylation of E2F1 by SETD6 creates a feedback loop in the regulation of SETD6 mRNA expression levels.

Further research is required to determine how SETD6 mRNA levels affect the malignant progression of prostate cancer. Through the identification of the precise mechanisms by which SETD6-mediated methylation of E2F1 modulates gene expression, it will become possible to develop novel approaches for the precise and targeted modulation of gene activity. This holds significant promise for the development of new therapies for a diverse range of diseases, including cancer and other disorders characterized by abnormal gene expression patterns.

## Experimental procedures

### Plasmids

The E2F1 sequence was amplified by PCR from a plasmid provided by Prof Assaf Rudich (BGU) and subcloned into the pcDNA3.1 3xFLAG plasmid using primers indicated in Table 1. For viral infections, E2F1 was cloned into the pWZL-FLAG plasmid (7). For recombinant protein purification, E2F1 was cloned into pET-SUMO and pGEX-6p1 plasmids (7). To generate E2F1 mutants, site-directed mutagenesis was performed using primers indicated in Table 1, followed by DNA sequencing for confirmation. All E2F1 lysine mutants were cloned into pET-SUMO, pGEX-6p1, pcDNA3.1 3xFLAG, and pWZL-FLAG plasmids. SETD6 promoter sequence was amplified and subcloned into pGL3 plasmid.

**Table 1:** Primers for cloning and mutagenesis

Name	Sequence (5′ to 3')
E2F1 Fw	TTAGGCGCGCCGCCTTGGCCGGGGCCCC
E2F1 Rev	GGCTTAATTAATCAGAAATCCAGGGGGGTGAGG
E2F1 K117R Fw	GCCGCCATCCAGGAAGAGGTGTGAAATCCCCG
E2F1 K117R Rev	CGGGGATTTCACACCTCTTCCTGGATGGCGGC
SETD6 promoter full-length Fw	TTAGGTACCAACCTCTATATTCACAGCCTC
SETD6 promoter full-length Rev	GGCCAAGCTTGTTCTGCGAACGGAGAAG

### Cell lines, transfections, infections, and treatments

Human embryonic kidney 293 and DU145 (purchased from American Type Culture Collection, kindly provided by Prof Etta Livneh, BGU) cells were maintained in Dulbecco’s modified Eagle’s medium (Sigma; D5671) with 10% fetal bovine serum (Gibco), penicillin–streptomycin (Sigma; P0781), 2 mM l-glutamine (Sigma; G7513), and nonessential amino acids (Sigma; M7145), at 37 °C in a humidified incubator with 5% CO_2_ as previously described (55). Cell transfection was performed using Mirus reagents (TransIT-LT1 or TransIT-X2), according to the manufacturer’s instructions. For the DU145 CRISPR–Cas9 SETD6 KO, two different guide RNAs for SETD6 (Table 2) were cloned into lentiCRISPR plasmid (Addgene; catalog no.: 49535). Following transduction and puromycin selection (2.5 μg/ml), single clones were isolated, expanded, and validated by sequencing.

**Table 2:** gRNAs for CRISPR–Cas9 KO cells

Name	Sequence (5′ to 3')
SETD6 gRNA #1	GGAGGCCCTACTTTGCGCTC
SETD6 gRNA #3	CTGGGATTTCCTATGCAAAC

### Recombinant proteins

*Escherichia coli* Rosetta strain (Novagen) was transformed with a plasmid expressing His-SUMO-/GST-E2F1 WT or K117R, His-SETD6 WT or Y285A mutant proteins, and the transformed cells were grown in LB medium. Bacteria were harvested by centrifugation after IPTG induction. The bacteria overexpressing His-tagged proteins were resuspended in lysis buffer containing 10 mM imidazole, 1 mM PMSF, and 0.1 v/v % Triton X-100 in PBS, incubated with 0.25 mg/ml lysozyme for 30 min on ice, and followed with lysis by sonication on ice (25% amplitude, 1 min total, 10/5 s ON/OFF). His-tagged proteins were purified using nickel–nitrilotriacetic acid beads (Pierce) or on a HisTrap column (GE) with the ÄKTA gel filtration system using PBS as wash buffer. Proteins were eluted by 0.5 M imidazole in PBS followed by dialysis to 10% glycerol in PBS. The bacteria overexpressing GST-E2F1 were resuspended in lysis buffer containing 50 mM NaCl, 5 mM EDTA, 0.15 mM PMSF, and 1% Triton X-100 in 50 mM Tris–HCl (pH 8). GST-E2F1 was purified on glutathione-sepharose 4B (GE) or on a GSTrap column (GE) with the ÄKTA gel filtration system using PBS as wash buffer. Proteins were eluted with 10 mg/ml reduced glutathione (Sigma) in 50 mM Tris (pH 8). Recombinant GST SETD6 was expressed and purified as previously described (7).

### Antibodies, WB analysis, and immunoprecipitation

Primary antibodies used were anti-FLAG (Sigma; catalog no.: F1804), antiactin (Abcam; catalog no.: ab3280), anti-Pan-Kme1 (Cell Signaling; catalog no.: 14679), anti-GST (Abcam; catalog no.: ab9085), anti-His (ThermoFisher Scientific; catalog no.: rd230540a), anti-SETD6 (Genetex; catalog no.: GTX629891), anti-E2F1 (SantaCruz; catalog no.: KH95), and anti-H3 (Abcam; catalog no.: ab10799). Horseradish peroxidase (HRP)–conjugated secondary antibodies, goat anti-rabbit, goat antimouse, and streptavidin–HRP were purchased from Jackson ImmunoResearch (catalog nos.: 111-035-144 and 115-035-062, respectively). For WB analysis, cells were homogenized and lysed in radioimmunoprecipitation assay buffer (50 mM Tris–HCl [pH 8], 150 mM NaCl, 1% Nonidet P-40, 0.5% sodium deoxycholate, 0.1% SDS, 1 mM DTT, and 1:100 protease inhibitor mixture [Sigma]). Samples were resolved on 8 to 12% SDS-PAGE, followed by WB analysis.

The polyclonal E2F1-K117me1 antibody was generated by Abmart, Inc using a GRHPGKme1GVK epitope identification peptide. For validation of its specificity, SPOT arrays were blocked in 5% milk in 1× Tris-buffered saline with Tween-20 solution for 1 h. Then, the array was incubated with the primary E2F1-K117me1 antibody solution (1:2000 dilution) overnight at 4 °C. The next day, the array was washed three times for 5 min with 1× Tris-buffered saline with Tween-20 solution incubated with the secondary antibody solution anti-rabbit HRP (Na934v; GE Healthcare; 1:5000 dilution) for 1 h at room temperature. After washing again, the signal was detected by chemiluminescence after the addition of Pierce ECL Western Blotting substrate.

For immunoprecipitation, proteins extracted from cells were incubated overnight at 4 °C with FLAG-M2 beads (Sigma; A2220) or with antibody of interest, to which Magna ChIP Protein A + G Magnetic Beads (Millipore; catalog no.: 16-663) were added for 2 h at 4 °C. The beads were then washed once with PBS and submitted to SDS-PAGE and WB analysis.

#### Synthesis of peptide SPOT arrays

Peptide arrays were generated by the SPOT synthesis method using the Autospot Multipep peptide array synthesizer (Intavis AG). Each peptide spot, with a diameter of 2 mm, contained approximately 9 nmol of peptide (Autospot Reference Handbook; Intavis AG). The successful synthesis of peptide arrays was verified by bromphenol blue staining. Each spot comprises 15-amino acid long peptides with different residues surrounding the potential methylation target site.

#### Methylation of peptide SPOT arrays

Peptide SPOT arrays were preincubated in methylation buffer containing 20 mM Tris–HCl (pH 9) and 5 mM DTT for 5 min on a shaker. Afterward, the SPOT arrays were incubated in methylation buffer containing additionally 50 nM SETD6 and 0.76 μM labeled [methyl-^3^H]-AdoMet (PerkinElmer, Inc) for 1 h at room temperature. Next, the arrays were washed five times for 5 min with 100 mM NH_4_HCO_3_ and 1% SDS. After washing, the arrays were incubated in Amplify NAMP100V solution (GE Healthcare) for 5 min. Then, the arrays were exposed to Hyperfilm high-performance autoradiography films (GE Healthcare) in the dark at −80 °C for different exposure times and developed using an Optimus TR developing machine.

#### *In vitro* protein methylation assay

About 1.6 μM of GST-tagged E2F1 WT or mutant was incubated with 0.2 μM of His-SUMO-tagged SETD6 WT or mutant in methylation buffer (20 mM Tris–HCl [pH 9] and 5 mM DTT), supplemented with 0.76 μM labeled [methyl-^3^H] -AdoMet (PerkinElmer) for 3 h at 25 °C. The reaction was stopped by the addition of SDS-PAGE loading buffer and heating for 5 min at 95 °C. Afterward, the samples were separated by 16% SDS-PAGE followed by the incubation of the gel in Amplify NAMP100V (GE Healthcare) for 1 h on a shaker. In the next step, the gel was dried for 2 h at 70 °C under vacuum. The signals of the transferred radioactively labeled methyl groups were detected by autoradiography using a Hyperfilm high performance autoradiography film (GE Healthcare) at −80 °C in the dark. The film was developed with an OptiMax Typ TR machine after different exposure times.

For the nonradioactive methylation assay, the reactions were supplemented with 1 mM of nonradioactive AdoMet (Sigma–Aldrich). The reaction was stopped by the addition of SDS-PAGE loading buffer and heated for 5 min at 95 °C. Afterward, the samples were separated by 16% SDS-PAGE followed by WB analysis.

### ELISA

His-SUMO-E2F1 (2 μg), MBP-RelA (2 μg), or BSA diluted in PBS were added to a 96-well plate (Greiner MICROLON) and incubated for 1 h at room temperature followed by blocking with 3% BSA for 30 min. Then, the plate was covered with 0.5 μg GST-SETD6 or GST protein (negative control) diluted in 1% BSA in PBS and Tween-20 for 1 h at room temperature. Plates were then washed and incubated with primary antibody (anti-GST; 1:4000 dilution) followed by incubation with HRP-conjugated secondary antibody (goat anti-rabbit; 1:2000 dilution) for 1 h. Finally, TMB reagent followed by 1 N H_2_SO_4_ (stop solution) was added; the absorbance at 450 nm was detected using Tecan Infinite M200 plate reader.

### RNA extraction and real-time qPCR

Total RNA was extracted using the NucleoSpin RNA Kit (Macherey–Nagel). Then, 200 ng of the extracted RNA was reverse transcribed to complementary DNA using the iScript cDNA Synthesis Kit (Bio-Rad) according to the manufacturer’s instructions. The real-time qPCR primers were designed using the universal probe library assay design center (Roche) and UCSC Genome Bioinformatics (Table 3). qPCR was performed using SYBR Green I Master (Roche) in a LightCycler 480 System (Roche) in a 384-well plate using the following cycling conditions: 5 min at 95 °C, 45 cycles of amplification; 10 s at 95 °C, 10 s at 60 °C, and 10 s at 72 °C, followed by melting curve acquisition; 5 s at 95 °C, 1 min at 65 °C, and monitoring up to 97 °C, and finally cooling for 30 s at 40 °C. All samples were amplified in four or five replicates. Gene expression levels were normalized relative to GAPDH gene and controls of the experiment.

**Table 3:** Primers for qPCR

Name	Sequence (5′ to 3')
Housekeeping gene	
GAPDH Fw	AGCCACATCGCTCAGACAC
GAPDH Rev	GCCCAATACGACCAAATCC
SETD6 gene	
SETD6 Fw	GGATGAAAAGGAGCCCAACT
SETD6 Rev	CTACCATCCGAAGACAATTCG

Abbreviations: Fw, forward; Rev, reverse.

### Chromatin extraction

Cells were crosslinked using 1% formaldehyde (Sigma) added directly to the medium and incubated on a shaking platform for 10 min at room temperature. The crosslinking reaction was stopped by adding 0.125 M glycine for 5 min. Cells were harvested and washed twice with PBS and then lysed in 0.5 ml cell lysis buffer (10 mM Hepes [pH 7.9], 10 mM KCl, 1.5 mM MgCl_2_, 340 mM sucrose, 10% glycerol, 0.1% Triton X-100, 100 nM PMSF, 1 mM DTT, and 1:200 protease inhibitor) for 8 min on ice. Samples were centrifuged (2000*g*, 5 min, 4 °C), and the pellets were washed with the aforementioned buffer, without protease inhibitor and centrifuged again. The nuclei pellets were lysed in 0.5 ml nuclei lysis buffer (3 mM EDTA, 0.2 mM EGTA, 1 mM DTT, and 1:200 protease inhibitor) for 30 min on ice. Samples were centrifuged (2000*g*, 5 min, 4 °C), and the chromatin pellets were solubilized in 200 μl resuspension buffer (10 mM Hepes [pH 7.9], 10 mM KCl, 1.5 mM MgCl_2_, 340 mM sucrose, 10% glycerol, 1:200 protease inhibitor, 1:200 benzonase nuclease enzyme [Sigma]) for 15 min at 37 °C.

For protein–protein interaction analysis, the soluble chromatin was precleared with Magna ChIP Protein A + G Magnetic Beads (Millipore; catalog no.: 16-663) for 1 h and then incubated overnight at 4 °C with magnetic FLAG-M2 beads or the indicated antibody, then A + G magnetic beads were added for 2 h at 4 °C. The immunoprecipitated complexes were washed once with PBS. Immunoprecipitated complexes in protein sample buffer were resolved in SDS-PAGE and analyzed by WB.

### Chromatin preparation and ChIP–qPCR

Cells were crosslinked using 1% formaldehyde (Sigma) added directly to the medium and incubated on a shaking platform for 10 min at room temperature. The crosslinking reaction was stopped by adding 0.125 M glycine for 5 min. Cells were harvested and washed twice with PBS and then lysed in 1 ml cell lysis buffer (20 mM Tris–HCl [pH 8], 85 mM KCl, 0.5% Nonidet P-40, and 1:100 protease inhibitor cocktail) for 10 min on ice. Nuclear pellets were resuspended in 200 μl nuclei lysis buffer (50 mM Tris–HCl [pH 8], 10 mM EDTA, 1% SDS, and 1:100 protease inhibitor cocktail) for 10 min on ice and then sonicated (Bioruptor, Diagenode) at high power settings for six cycles, 6 min each (30 s ON/OFF). Samples were centrifuged (20,000*g*, 15 min, 4 °C), and the soluble chromatin fraction was collected. The chromatin fraction was diluted five times in dilution buffer (20 mM Tris–HCl [pH 8], 2 mM EDTA, 150 mM NaCl, 1.84% Triton X-100, and 0.2% SDS). The chromatin fraction was precleared overnight at 4 °C with A + G magnetic beads. The precleared sample was then immunoprecipitated with magnetic FLAG-M2 beads or A + G magnetic beads preconjugated with the indicated antibody. The immunoprecipitated complexes were washed according to the chromatin extraction protocol detailed previously. DNA was eluted with elution buffer (50 mM NaHCO_3_, 140 mM NaCl, and 1% SDS) containing ribonuclease A (0.2 μg/μl) and proteinase K (0.2 μg/μl). Finally, the DNA eluates were decrosslinked at 65 °C overnight with shaking at 900 rpm and purified by NucleoSpin Gel and PCR Clean-up kit (Macherey–Nagel), according to the manufacturer’s instructions.

Purified DNA was subjected to qPCR using specific primers (Table 2). Primers were designed based on E2F1 occupancy (in correlation with H3K4me3, H3K27Ac, ATAC-Seq, and TF clusters in *SETD6* promoter locus) found in different ChIP-Seq data previously published in the National Center for Biotechnology Information GEO datasets by Ramos-Montoya *et al.* (56) (GEO accession: GSM1207898), Barfeld *et al.* (57) (GEO accessions: GSM1907203 and GSM1907213), Bert *et al.* (58) (GEO accession: GSM947524), and Liu *et al.* (59) (GEO accession: GSM2186480) and viewed using Integrated Genomics Viewer software (60). qPCR was performed using SYBR Green I Master (Roche) in a LightCycler 480 System (Roche). All samples were amplified in four replicates in a 384-well plate using the following cycling conditions: 5 min at 95 °C, 45 cycles of amplification; 10 s at 95 °C, 10 s at 60 °C, and 10 s at 72 °C, followed by melting curve acquisition; 5 s at 95 °C, 1 min at 65 °C, and monitoring up to 97 °C, and finally cooling for 30 s at 40 °C. The results were normalized to input DNA and presented as percent input. Primers used for the ChIP–qPCR are listed in Table 4.

**Table 4:** Primers for ChIP–qPCR

Name	Sequence (5′ to 3')
SETD6 promoter peak Fw	CAGCATGCTACTTCCCCAGG
SETD6 promoter peak Rev	CCTCTCTGCCCTTATTTGCCAG
SETD6 promoter NC Fw	CACTCACCCGTGGACGCTTC
SETD6 promoter NC Rev	CTGCAGCGTGGACGACAAAAC
ChIP NC Fw	GTGCCTCCCAAAGCTGAGAT
ChIP NC Rev	GATTTCTGGGTGACTGGGCA

Abbreviations: Fw, forward; NC, negative control; Rev, reverse.

### Dual-luciferase assay

DU145 cells were seeded in 24-well plates and transiently transfected with 0.1 μg FLAG-E2F1 WT or K117R mutant or 25 mM siRNA, 0.1 μg firefly luciferase plasmid, containing SETD6 promoter variants, and 0.1 μg Renilla luciferase plasmid. Total amount of transfected DNA in each dish was kept constant by the addition of empty vector, as necessary. Cell extracts were prepared 30 h after transfection, and firefly luciferase activity was measured with the Dual-Glo Luciferase Assay system (Promega) and normalized to that of Renilla luciferase. Luminescence was measured by Tecan Infinite M200 plate reader.

### Statistical analyses

Statistical analyses for all assays were analyzed with GraphPad Prism software (GraphPad Software, Inc), using Student’s two-tailed *t* test (unpaired) or one-way ANOVA with a Tukey's post hoc test.

## Data availability

All the data supporting our findings are contained within the article.

## Supporting information

This article contains supporting information.

## Conflict of interest

The authors declare that they have no conflicts of interest with the contents of this article.

## Supporting information

Supporting Table S1 E2F1 predicted binding sites. E2F1 binding sites at the SETD6 promoter sequence, as predicted by the JASPAR database (https://jaspar.genereg.net/) with relative profile score threshold of 90%. The prediction is based on curated transcription factors binding profiles as position frequency matrices (PFMs).
