# Supplementary figures and images for: Methylation of the transcription factor E2F1 by SETD6 regulates SETD6 expression via a positive feedback mechanism
Source: J Biol Chem. 2023 Sep 9;299(10):105236. doi: 10.1016/j.jbc.2023.105236 (PMC10551896; doi:10.1016/j.jbc.2023.105236)

**
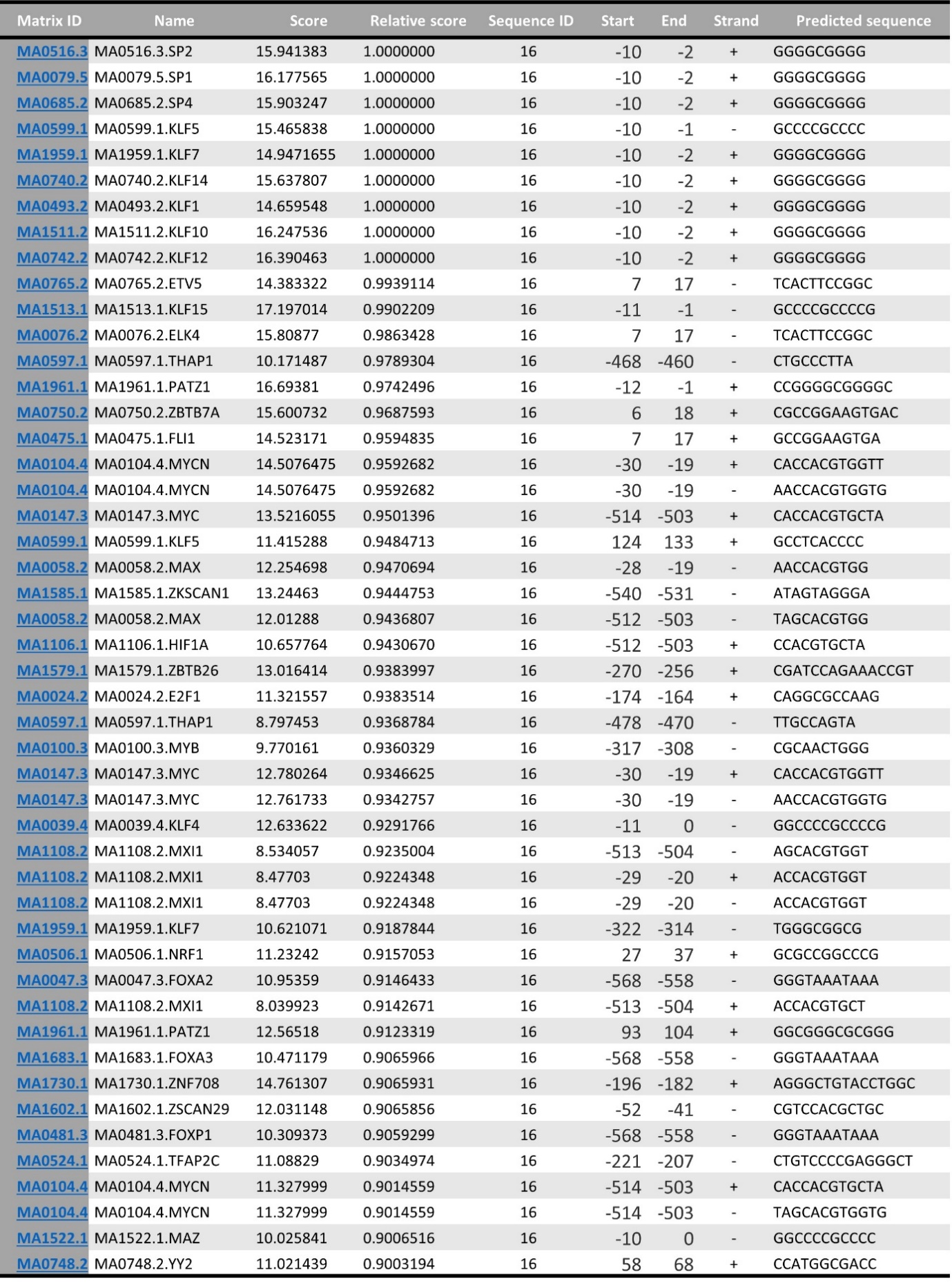
**

Supplement: Supporting Table S1 — E2F1 predicted binding sites. E2F1 binding sites at the SETD6 promoter sequence, as predicted by the JASPAR database (https://jaspar.genereg.net/) with relative profile score threshold of 90%. The prediction is based on curated transcription factors binding profiles as position frequency matrices (PFMs). [file mmc1.docx]
